# Autonomic neuropathy in diabetes

**DOI:** 10.20945/2359-4292-2025-0406

**Published:** 2025-11-24

**Authors:** Lucianne Righeti Monteiro Tannus, Roberta Arnoldi Cobas

**Affiliations:** 1 Departamento de Clínica Médica, Unidade de Diabetes, Universidade Estadual do Rio de Janeiro, Rio de Janeiro, RJ, Brasil

**Keywords:** Diabetic neuropathy, diabetic autonomic neuropathy, cardiovascular autonomic neuropathy, diabetes mellitus, heart rate variability

## Abstract

Diabetic autonomic neuropathy (DAN) is a serious and often under-recognized complication of diabetes that can affect any division of the autonomic nervous system (ANS), presenting with a wide range of signs and symptoms. The pathophysiology of DAN involves a complex interplay of hyperglycemia-driven metabolic and vascular pathways, oxidative stress, inflammation, and autonomic imbalance, ultimately leading to progressive nerve dysfunction. Cardiovascular autonomic neuropathy (CAN) has emerged as a particularly severe condition, associated with heightened risk of arrhythmia, silent myocardial ischemia, heart failure, and mortality. DAN, however, extends beyond the cardiovascular system, encompassing gastrointestinal (GI), genitourinary (GU), and sudomotor dysfunctions, that strongly impair quality of life. Despite its impact, DAN remains largely overlooked in clinical practice due to its subclinical onset, non-specific symptoms, and limited routine screening. This review integrates basic, epidemiological, and clinical data to provide a practical understanding of DAN with the aim of helping clinicians to suspect, investigate and manage DAN, with particular attention to its cardiovascular (CV), GI, and GU manifestations.

## INTRODUCTION

Diabetic autonomic neuropathy (DAN) is a common and serious condition (^1^,^2^). DAN is defined as ‘a disorder of the autonomic nervous system (ANS) occurring in the context of diabetes or metabolic disturbances associated with prediabetes, after the exclusion of other causes’ according to the Toronto Consensus (^3^). Despite its impact on quality of life and morbimortality, DAN remains underrecognized and poorly understood (^4^).

The ANS comprises two main branches, the sympathetic (SNS) and parasympathetic (PNS) nervous systems. Although functionally antagonistic, they act in coordination to maintain homeostasis. SNS activation triggers “fight-or-flight” responses, such as increased heart rate (HR), blood pressure (BP), energy mobilization, and alertness. In contrast, PNS activation counterbalances these effects, slowing HR, reducing cardiac contractility, and promoting digestion (^5^). This dynamic balance enables adaptation to physiological demands and its disruption may lead to maladaptive responses in multiple organ systems, sometimes detectable only by specific tests (^3^).

This review integrates basic, epidemiological, and clinical data to provide a practical understanding of DAN with the aim of helping clinicians to suspect, investigate and manage DAN, with particular attention to its cardiovascular (CV) gastrointestinal (GI), and genitourinary (GU) manifestations.

## EPIDEMIOLOGY AND RISK FACTORS

The prevalence of cardiovascular autonomic neuropathy (CAN) is highly variable according to the studied population and the diagnostic criteria applied (^6^,^7^). It can manifest early in the onset of diabetes and its prevalence increases with longer duration, older age and worse glycemic control (^6^,^7^). Overall, CAN affect ~20% of individuals with diabetes and up to 65% of older individuals with long-standing disease (^8^,^9^). In prediabetes, data are limited, but the KORA study indicate a stepwise increase with worsening glycemic status: 4.5% in normoglycemia, 5.9% in impaired glucose tolerance (IGT), 8.1% in impaired fasting glucose (IFG), and 11.4% in combined IFG and IGT (^10^).

In The Anglo-Danish-Dutch Study of Intensive Treatment in People With Screen-Detected Diabetes in Primary Care (ADDITION), including 299 participants with type 2 diabetes (T2D), CAN prevalence increased from 9% at 6 years to 15% at 13 years, corresponding to an annual incidence of 1.8% (^11^), lower than previously reported of 4.6%-6% per year (^9^,^12^), probably reflecting early diagnosis and intensive treatment (^8^,^11^). In the SEARCH study (^13^), prevalence was 12% in youth with type 1 diabetes (T1D), and 17% in those with T2D, higher than in ADDITION study (^11^), partly due to methodological differences in CAN assessment.

In the Diabetes Control and Complications Trial/Epidemiology of Diabetes Interventions and Complications (DCCT/EDIC) study in individuals with T1D, CAN prevalence increased from 5% at baseline to 44% after 23 years of follow-up (^14^).

In a Brazilian multicenter study, including 1,712 individuals with T1D, CAN prevalence was 23.4% (^15^). Traditional risk factors included older age, poor glycemic control, diabetic kidney disease (DKD), diabetic retinopathy (DR), hypertension, elevated cholesterol levels and smoking (^15^), as well as emerging risk factors, such as lower socioeconomic status and poorer healthrelated quality of life (^15^). Glycemic variability and psychological conditions such as depression have been associated with the presence of CAN (^16^).

GI autonomic neuropathy affects 30%-50% of individuals with diabetes (^17^-^19^). Gastroparesis is the most common clinical manifestation (^6^), though confirmed prevalence remains relatively low. However, gastroparesis-related hospitalizations have increased, partly due to greater use of motilityimpairing agents, including opioids, GLP1 receptor agonists (GLP1-RA), and recreational marijuana (^1^,^6^). A meta-analysis reported a global prevalence of 9.3% (4.6% in women and 3.4% in men), higher in individuals with T2D (12.5%) than T1D (8.3%) (^20^).

GU dysfunction encompasses bladder and sexual dysfunction, as well as recurrent urinary tract infections (UTI) (^18^,^21^,^22^). A meta-analysis including 67,040 individuals with diabetes, reported a pooled prevalence of sexual dysfunction of 61.4% (95% CI: 51.8%-71.0%), with substantial heterogeneity (I^2^ = 71.6%) (^22^). In the National Health and Nutrition Examination Survey (NHANES), lower urinary tract symptoms (LUTS) (nocturia, hesitancy, or incomplete emptying) were more frequent in individuals with diabetes compared with the overall population (52.7 *vs.* 36.5%) (^23^). In women with diabetes, LUTS prevalence ranges from 24% to 49% (^24^). In the DCCT/EDIC, moderate-to-severe LUTS developed in ~20% of men with T1D after 10 years of follow-up (mean diabetes duration 22.1 years) (^25^). Among women, any and at least weekly urinary incontinence (UI) was reported in 38% and 17%, respectively, with advancing age, higher body weight, and prior UTI as risk factors (^26^).

## PATHOGENESIS OF DAN

Diabetic neuropathy (DN), including DAN, results from multifactorial mechanisms in which oxidative stress and inflammation play central roles, leading to neuronal damage, endothelium dysfunction and neural ischemia (^19^,^27^,^28^).

Data from the DCCT (^29^) and United Kingdom Prospective Diabetes Study (UKPDS) (^30^) indicate that hyperglycemia is the main driver, with additional contributions from dyslipidemia, hypertension, genetic predisposition, obesity (particularly in T2D), low insulin and C-peptide levels, and autoimmunity (notably in T1D) (^7^,^27^,^28^).

Over the next lines we will summarize the intracellular molecular mechanisms involved in neural and endothelial damage.

Chronic hyperglycemia increases intracellular glucose, accelerating glycolysis and electron flux in the mitochondrial electron transport chain (^27^,^28^). This enhances mitochondrial production of reactive oxygen species (ROS), particularly superoxide, leading to oxidative stress, mitochondrial dysfunction, reduced ATP generation, and neuronal apoptosis (^19^,^27^,^28^). ROS-induced mitochondrial DNA damage activates poly (ADP-ribose) polymerase (PARP), which inhibits glycolysis via glyceraldehyde-3-phosphate dehydrogenase (GAPDH) inactivation (^7^,^27^), causing accumulation of upstream glycolytic intermediates.

These compounds are redirected into four key major pathways - the polyol, the advanced glycation endproduct (AGE), the hexosamine and the protein kinase C (PKC) pathways - resulting in oxidative injury, reduced nitric oxide (NO) bioavailability and endothelial dysfunction, sorbitol-induced osmotic stress, neuroinflammation, and ultimately nerve damage with demyelination and impaired nerve conduction (^19^,^27^,^28^).

Insulin signaling supports nerve metabolism and function via PI3K mediated ATP production (^31^) while impaired signaling affects T1D and T2D differently: in T2D, insulin resistance limits metabolic and bioenergetic function, and compromise axonal growth and myelination in peripheral nerves (^19^,^32^), whereas in T1D, preserved insulin sensitivity allows better neuropathic outcomes with insulin therapy (^33^). Hyperglycemia further inhibits the PI3K/Akt signaling pathway in Schwann cells, leading to apoptosis, demyelination, and impaired nerve conduction (^34^).

Defects in the interactions between afferent and efferent autonomic local neural pathways are involved in the pathogenesis of GI and GU autonomic neuropathy. As a result, GI motor, sensory and secretory functions may be impaired (^35^) as well as bladder sensation, capacity and urinary retention (^21^).

Endothelial dysfunction besides autonomic nerve damage may lead to reduced cavernosal smooth muscle relaxation, altered sensory function, and impaired motor control of erectile muscles in men and decreased arousal and inadequate lubrification in women (^21^,^24^).

In the early stages of T2D, CAN is marked by sympathovagal imbalance (^8^,^36^,^37^). Insulin resistance and compensatory hyperinsulinemia drive sustained SNS overactivation (^6^,^8^), through endothelium-dependent vasodilation, baroreflex activation, hypothalamic pathways, and chemoreceptor sensitization (^6^-^8^,^37^). DN progresses in a length-dependent manner, making the vagus nerve - the longest autonomic nerve responsible for ~75% of PNS activity - particularly vulnerable. Its early involvement reduces PNS tone, and reinforces SNS predominance (^8^,^37^).

Beyond the contribution of inflammation to DAN, autonomic dysfunction itself may exacerbate inflammatory processes (^38^). In CAN, vagal impairment reduces cholinergic anti-inflammatory signaling, while SNS overactivity enhances macrophage activation and cytokine release, perpetuating inflammation (^38^-^41^). These alterations in the inflammatory reflex suggest a role of the ANS in modulation immune and inflammatory responses (^39^-^41^,^42^).

In summary, the pathophysiology of DAN is complex and multifactorial, involving metabolic, vascular, inflammatory, neurotrophic, and emerging molecular mechanisms. Despite significant advances in understanding its underlying pathways, further studies are needed to fully elucidate the intricate processes involved and to identify effective and targeted therapeutic strategies.

## CLINICAL SPECTRUM, DIAGNOSIS AND MANAGEMENT OF DAN

**Figure 1:**
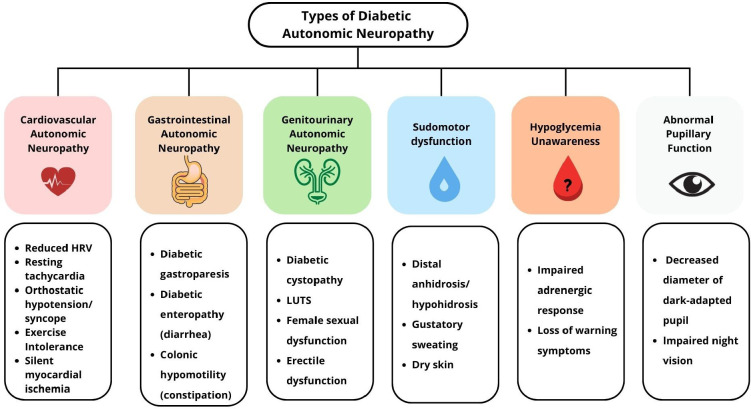
Clinical spectrum of diabetic autonomic neuropathy.Diabetic autonomic neuropathy may affect multiple systems, including cardiovascular, gastrointestinal, genitourinary, and sudomotor pathways, as well as pupillary function and hypoglycemia awareness. Each subtype presents with characteristic symptoms that reflect widespread autonomic dysfunction in diabetes.

HRV: heart rate variability; LUTS: lower urinary tract symptoms.

DAN encompasses a broad spectrum of manifestations involving CV, GI, and GU systems, among others (**Figure 1**). Symptomatic forms are relatively uncommon (^43^), except for erectile dysfunction (ED), which is multifactorial, and GI symptoms, which are frequent in the general population and not strongly associated with objective GI motor dysfunction or cardiovascular autonomic reflex tests (CARTs) abnormalities (^43^).

### Cardiovascular Autonomic Neuropathy CAN

#### and Other Microvascular Complications

CAN often precedes albuminuria (^44^,^45^) and independently predicts both the development and progression of DKD in individuals with diabetes (^42^,^46^,^47^). Proposed mechanisms include sympathovagal imbalance, nocturnal SNS hyperactivity increasing BP and glomerular pressure (^9^,^48^), enhanced tubular reabsorption via renal SNS innervation, angiotensin-mediated microvascular injury, and erythropoietin-deficiency anemia (^47^,^49^). In advanced stages, daytime orthostatic hypotension (OH), may further impair renal hemodynamics (^9^,^50^). In the Atherosclerosis Risk in Communities (ARIC) study (n = 13,241 adults; 11.5% with diabetes), higher resting HR and lower heart rate variability (HRV) increased risk of end-stage renal disease and chronic kidney disease related hospitalizations over 16 years (^47^).

CAN is also linked to peripheral diabetic neuropathy (PDN) and DR development and progression (^15^,^51^-^53^). A systematic review demonstrated that the PDN presence and severity was correlated with worse autonomic outcomes, suggesting shared pathophysiological mechanisms (^54^). Prospective data from a study involving 725 adolescents with T1D, and follow-up of 3.8 years, demonstrated that CAN predicted the incidence of DR and early kidney dysfunction, even after adjusting for glycated hemoglobin (A1c) and diabetes duration (^53^). Likewise, in a 3-year cohort study of 4,850 adults with T2D, lower high-frequency (HF) power, a marker of autonomic dysfunction, was an independent predictor of DR progression, alongside mean A1c and baseline proliferative DR (^55^).

These findings underscore the importance of integrated, systematic screening of microvascular complications in diabetes.

#### CAN and its impact on cardiovascular morbidity and mortality

Evidence from the Framingham Offspring Study indicates that autonomic imbalance may precede diabetes onset (^50^). In 1,882 participants, higher resting HR and lower HRV, sex, age, and smoking, predicted hypertension, hyperglycemia, diabetes diagnosis, cardiovascular disease (CVD) and mortality over 12 years (^50^). These findings suggest a bidirectional relationship between autonomic dysfunction and glucose abnormalities that warrants further investigation.

CAN also contributes to myocardial dysfunction, left ventricular (LV) hypertrophy and heart failure (^56^). Diastolic dysfunction, the early manifestation of diabetic cardiomyopathy, manifests as impaired ventricular relaxation and filling (^9^,^56^,^57^). In the DCCT/EDIC, CAN was associated with increased LV mass and mass-to-volume ratio, markers of concentric remodeling (^56^).

CAN may also impair coronary flow autoregulation, lowering the threshold for myocardial ischemia, and increasing the risk of silent myocardial ischemia (SMI) and infarction. Multiple studies confirm the strong association between CAN and CV risk and mortality in both T1D and T2D (^8^,^9^,^57^-^59^). In the Detection of Silent Myocardial Ischemia in Asymptomatic Diabetic Subjects (DIAD) study, including 1,123 asymptomatic individuals with T2D, abnormal Valsalva response (OR 5.6), longer diabetes duration (OR 5.2) and male sex (OR 2.5) independently predicted SMI over 5 years, whereas traditional risk factors failed to predict its occurrence (^60^).

In the Action to Control Cardiovascular Risk in Diabetes (ACCORD) trial, involving 8,135 participants with T2D, CAN was associated with a 1.55 to 2.14fold higher risk of mortality, independent of baseline CVD, diabetes duration, and other risk factors (^61^). Likewise, in the EURODIAB Study of 2,787 individuals with T1D, CAN [HR 2.40 (1.32-4.36)] was one of the strongest predictor of all-cause mortality, alongside macroalbuminuria [2.39 (1.19-4.78)] and PDN [1.88 (1.06-3.35)] (^62^). A meta-analysis of 15 studies confirmed increased mortality risk, particularly when CAN was defined by ≥ 2 abnormal tests [relative risk 3.45 (95% CI: 2.66-4.47) compared with only one abnormality [1.20 (95% CI: 1.02-1.41)] (^63^).

SNS cardiac denervation is an important prognostic marker in heart failure and ischemic cardiomyopathy. In The AdreView Myocardial Imaging for Risk Evaluation in Heart Failure (ADMIRE-HF), reduced cardiac SNS innervation, assessed by I-123 mIBG scintigraphy (abnormal heart-to-mediastinum ratio < 1.6), predicted heart failure progression, arrhythmic events and cardiac death in 961 individuals with New York Heart Association (NYHA) class II/III heart failure and LV ejection fraction ≤ 35% (^64^). Similarly, in the Prediction of ARrhythmic Events with Positron Emission Tomography (PAREPET) study, SNS denervation, measured by 11C-HED PET, strongly predicted sudden cardiac arrest in patients with ischemic cardiomyopathy (^65^). These findings highlight that, beyond cardiovagal impairment, SNS denervation predicts cause-specific mortality from sudden cardiac death (^57^).

CAN has also been linked to increased risk of stroke (^9^,^57^,^58^), arterial stiffness (^66^), perioperative morbidity and mortality, due to hemodynamic instability (^9^,^58^,^67^), and sudden cardiac death, often associated with arrhythmias and QT interval prolongation (^68^,^69^).

The relationship between CAN and impaired awareness of hypoglycemia (IAH) remains complex. IAH, characterizes by reduced perception of hypoglycemia and blunted adrenergic responses, increases the risk of severe hypoglycemia (^70^). Although CAN has been associated with severe hypoglycemia in studies such as ACCORD (^70^-^72^), evidence indicates that even a single hypoglycemic episode may trigger IAH, and improvements are possible regardless of autonomic status, as shown in HypoCOMPaSS (^71^,^73^-^75^). Thus, while CAN may exacerbate IAH, it is not considered its primary determinant (^2^).

In summary, CAN is a major contributor to excess CV risk and mortality in diabetes, independent of traditional risk factors.

#### Clinical presentation of CAN

**Figure 2:**
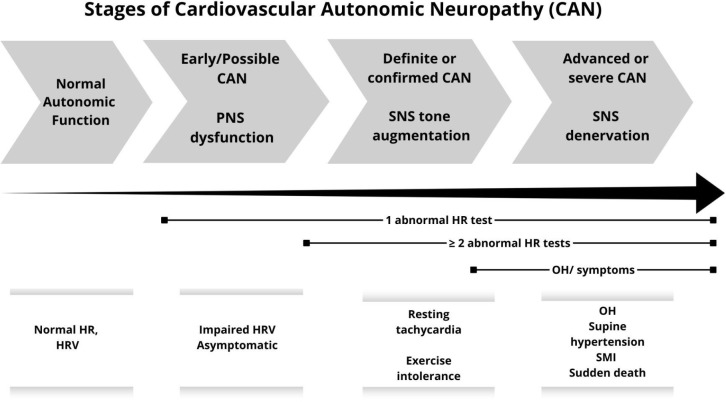
Stages of cardiovascular autonomic neuropathy.

ANS: autonomic nervous system; CAN: cardiovascular autonomic neuropathy; CARTs: cardiovascular autonomic reflex tests; HR: heart rate; HRV: heart rate variability; OH: orthostatic hypotension; PNS: parasympathetic nervous system; SMI: silent myocardial ischemia; SNS: sympathetic nervous system.

CAN is frequently underdiagnosed because early stages are often asymptomatic (^1^,^8^,^40^,^59^) (**Figure 2**). At this stage, it can only be detectable by reduced HRV, making early diagnosis challenging (^1^,^6^,^8^,^9^,^19^,^38^). Initial clinical manifestations include resting tachycardia (up to 130 beats/min). With worsening autonomic dysfunction, chronotropic and inotropic impairment can result in a fixed HR, often manifesting as exercise intolerance (^8^,^9^,^40^).

Diabetes affect ANS in an ascending length-dependent manner, with the vagus nerve usually being the first to be affected. CAN evolves from a subclinical stage, characterized by HRV abnormalities, to a clinical stage where PNS dysfunction leads to abnormal CARTs and symptoms of SNS predominance. Symptomatic CAN generally appears in the later stages, when cardiac involvement becomes more evident and progression is further marked by SNS denervation, orthostatic intolerance, and increased cardiovascular risk.

Advanced autonomic dysfunction may cause dizziness, unsteadiness, syncope and circadian BP dysregulation (nonor reverse-dipping), raising nocturnal CV risk (^48^,^76^). Importantly, while resting tachycardia may indicate CAN, its absence does not exclude the diagnosis.

OH, defined as a fall in systolic blood pressure (SBP) ≥ 20 mmHg (or ≥ 30 mmHg in individuals with hypertension) and/or diastolic blood pressure (DBP) ≥ 10 mmHg (or ≥ 15 mmHg in individuals with hypertension) within 3 minutes of standing (^8^,^77^), results from efferent SNS vasomotor denervation, leading to impaired splanchnic and peripheral vasoconstriction. Additionally, a blunted HR response and reduced cardiac output further contribute to the development of orthostatic symptoms (^77^,^78^).

In the EURODIAB IDDM Complications Study, including 3,007 individuals with T1D, OH prevalence was 5.9%, 18%, and 32%, depending on definition (a fall in SBP >30, >20, and >10 mmHg, respectively), with 18% reporting orthostatic symptoms (^79^). These symptoms are often worse in the morning, after meals, with heat, prolonged standing, or exercise (^80^) and increases the risk of fall.

Medical guidelines recommend screening all patients with diabetes for autonomic symptoms as the first step in DAN evaluation (^7^,^9^). However, as symptoms typically appear late and are nonspecific, their utility for early diagnosis is limited. Standardized questionnaires, such as the Composite Autonomic Symptom Score 31 (COMPASS 31) (^81^) and the Survey of Autonomic Symptoms (SAS) (^82^,^83^) address multiple autonomic domains, and provide practical, low-cost tools. COMPASS 31 shows fair diagnostic accuracy for CAN (AUC 0.75, sensitivity of 75% and 70% and specificity of 65% and 67% for global and confirmed CAN, respectively) (^84^). Strong diagnostic performance was also observed with SAS, which demonstrated an AUC of 0.828 (^82^).

However, evidence suggests that the diagnostic performance of COMPASS 31 may vary according to diabetes type. In a study evaluating 79 individuals with T1D and 140 individuals with T2D (^85^), the COMPASS score was significantly associated with confirmed CAN in T1D but not in T2D. Correlations between COMPASS 31 and CARTs were also observed only in T1D. Moreover, COMPASS 31 demonstrated higher sensitivity in T1D (81.2%) compared to T2D (67.7%) and fair diagnostic accuracy for confirmed CAN in T1D but not in T2D (AUC 0.61) (^85^). These differences should be considered when applying COMPASS 31 in clinical practice.

In addition to symptom-based questionnaires, clinical risk scores are being developed to support CAN detection in clinical practice. In a retrospective cross-sectional study, including 115 individuals with T1D and 161 with T2D, standard CARTs were performed and a CAN risk score was developed based on the strength of associations between clinical variables and confirmed CAN (defined by the presence of ≥ 2 abnormal CARTs) (^86^). The score incorporated variables such as resting HR, A1c, DR, DKD and CVD in both types of diabetes, with additional parameters including HDL cholesterol, SBP, and smoking in T1D, and insulin treatment and physical activity in T2D. The CAN risk score demonstrated high diagnostic accuracy, with an AUC of 0.890 in T1D and 0.830 in T2D. Using a cut-off of 4, sensitivity was 88% in T1D and 78.6% in T2D, specificity 74.4% and 73.5%, and negative predictive value 95.7% and 97.3%, respectively (^86^). Although promising, these scores still require external validation before being adopted as universal screening tools.

#### CAN diagnosis

Assessment of CAN in clinical settings combines the evaluation of autonomic signals and symptoms with standardized tests of CV autonomic regulation (^8^). Because several comorbidities and medications may mimic autonomic dysfunction, careful exclusion of these conditions is essential before confirming CAN (^7^). Resting tachycardia (HR > 100 bpm) can result from anemia, hyperthyroidism, dehydration, smoking, CVD, or the use of sympathomimetic, ephedrine or pseudoephedrine, dietary supplements, alcohol, caffeine or recreational drugs (^7^). OH, in turn, may instead reflect intravascular volume depletion, adrenal insufficiency, CVD or the use of diuretics, antihypertensives, sedatives, anticholinergics, neuroleptics and other drugs (^7^).

In clinical practice, tachycardia, OH, QT prolongation and abnormal 24 h ambulatory BP monitoring (ABPM) patterns are non-invasive, widely available tools that may provide early clues to CAN before specialized testing (^8^,^9^,^87^). The Toronto Consensus recommends routine screening for orthostatic symptoms and annual OH testing in all patients with diabetes, particularly those > 50 years or with hypertension (^9^).

ABPM is useful to detect nondipping (nocturnal BP fall < 10%) or reverse dipping patterns (no decline or a rise) (^8^) which may guide antihypertensive treatment (^9^). However, the day-night SBP difference shows high specificity (95%) but low-sensitivity (25%) as a marker of CAN (^88^).

Similarly, a prolonged QT interval has high specificity (86%) but low-sensitivity (28%) for CAN diagnosis (^89^). Rather than a screening tool, it aids arrhythmic risk stratification and predicts mortality, thus being more relevant for prognostic evaluation (^8^,^43^,^59^,^69^).

According to the Toronto Consensus (^9^), CARTs are the gold standard for CAN assessment, being sensitive, specific, reproducible, safe, and standardized (^9^,^59^). CARTs assess autonomic CV function through physiological maneuvers that induce HR and BP oscillations (^9^,^43^,^90^). Ewing’s battery includes HR responses to deep breathing, Valsalva maneuver, and lying-to-standing, which assess PNS function, while BP responses to standing (OH) or sustained handgrip, reflects SNS activity (^9^,^91^,^92^). The latter is no longer recommended due to technical difficulty, low sensitivity, limited reproducibility, and risk of hemodynamic stress (^8^,^9^,^93^).

There is no evidence that one autonomic test is superior, nor consensus on the exact number of tests required for CAN diagnosis (^58^). However, most guidelines recommend more than one autonomic test to reduce the likelihood of false positive results (^9^,^43^,^94^). CAN staging is based on the number of abnormal CARTs: one suggests early or possible CAN, two confirm the diagnosis, and HO indicates advanced disease, associated with increased morbidity and mortality (^9^,^77^,^90^).

Given their physiological basis, CARTs must be standardized, with attention to confounding factors and proper instructions for performance and interpretation (^9^,^43^). Caffeine, alcohol, nicotine and drugs affecting ANS function should be avoided prior to testing, as well as strenuous exercise within 24 h (^9^,^43^). CARTs should be performed at fasting or at least 2 h after a light meal, with adequate glycemic control, and postponed in the presence of acute conditions such as fever, infection, dehydration, or significant emotional stress, given their potential to transiently alter CV autonomic regulation (^9^,^43^).

Interpretation should be based on normal agerelated reference values due to the physiological decline of HRV with aging (^43^). In the lying-to-standing test, approximately 25% of the SBP drop depends on baseline supine SBP, with greater decreases observed at > 160 mmHg and blunted responses at < 120 mmHg, potentially causing false-positive or negative results, respectively (^43^). Additionally, the presence of cardiac arrhythmias or the presence of a pacemaker invalidate the performance of CARTs (^43^), and the Valsalva maneuver should be avoided in individuals with proliferative DR due to the small risk of intraocular hemorrhage or lens dislocation (^92^).

However, CARTs are not widely available, and shortterm (5-minute) HRV analysis has been investigated as a simpler alternative (^92^,^95^). HRV is obtained from resting electrocardiogram (ECG) recordings, ideally under paced breathing, and analyzed by specific software that measures beat-to-beat (RR) intervals, providing timeand frequency-domain indices of sympathovagal integrity and balance. Time domain measures include RR mean, the difference between the longest and shortest RR intervals, the standard deviation of all normalto-normal RR intervals (SDNN; a measure of both SNS and PNS activity) and root mean square of successive differences between RR intervals (RMSSD; a primarily measure of PNS activity) (^5^,^58^). Longer recordings also allow calculation of the percentage of consecutive RR intervals differing > 50 ms (pNN50) (^87^).

**Figure 3:**
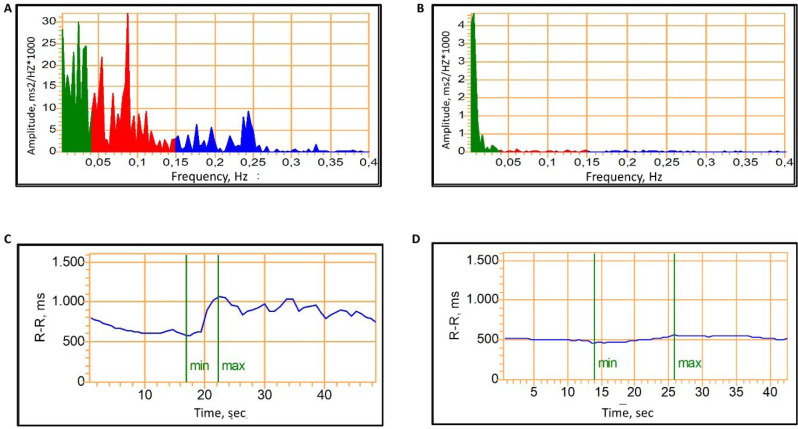
Example of spectral analysis study of HRV and CART in individuals with and without cardiovascular autonomic neuropathy.

CAN: cardiovascular autonomic neuropathy; CARTs: cardiovascular autonomic reflex tests; HF: high frequency; HR: heart rate; HRV: heart rate variability; LF: low frequency; PNS: parasympathetic nervous system; RR: RR interval; SNS: sympathetic nervous system; VLF: very low frequency.

In frequency domain (spectral) HRV analysis, electrocardiographic signals from sequential RR intervals are processed by algorithms to decompose the signal into its frequency components (^9^,^43^,^58^). Results are displayed as an amplitude-frequency diagram, showing oscillation magnitude (HR fluctuations per second) as a function of frequency (hertz). Three main components are distinguished in spectral analysis: very low frequency (VLF, 0.01-0.04 Hz), reflecting vasomotor tone fluctuations related to thermoregulation and sweating, predominantly under SNS control; low frequency (LF, 0.04-0.15 Hz), associated with the baroreflex, reflecting both SNS and PNS modulation; and high frequency (HF, 0.150.40 Hz), corresponding to PNS activity (^87^,^95^,^96^) (**Figure 3**). Short-term recordings cannot estimate long RR intervals fluctuations, such as the VLF component (^97^). Hence, apart from the VLF, LF and HF components provide information on both autonomic branches, and abnormal results may indicate impaired HRV, while the LF/HF ratio is commonly used as an index of sympathovagal balance (^87^,^96^,^98^).

Panels A and B depict HRV amplitude plotted as Amplitude (Y axis) against Frequency (X axis), derived from spectral analysis. This method reflects both the magnitude of HRV and its frequency-specific oscillations, enabling a distinction between the relative contributions of the SNS and PNS: blue corresponds to the HF band (0.15-0.40 Hz), which reflects PNS activity; red to the LF band (0.04-0.15 Hz), which is associated with baroreflex activity; and green to the VLF band (0.01-0.04 Hz), which reflects vasomotor tone fluctuations related to thermoregulation and sweating, predominantly under SNS control. Panel C and D depict the HRV response expressed in milliseconds over time during the performance of the Valsalva maneuver (an example of CART). In **Panel A**, a greater oscillation amplitude is observed in all three spectral bands (patient without CAN), whereas patients with CAN show a reduced amplitude in HRV, demonstrating impaired HRV (**Panel B**). **Panel C** illustrates the results of a Valsalva maneuver in a patient without CAN, demonstrating an expected reduction in RR interval during forced expiration followed by an expected increase in RR interval after release, reflecting an appropriate autonomic response. In contrast, this variability is absent in patients with CAN (**Panel D**).

HRV tests are sensitive, and require no active patient cooperation, but they may not necessarily identify CAN in same individuals as CARTs. Impaired HRV indices can precede CARTs abnormalities (^87^). However, HRV has low reproducibility and is prone to technical artifacts and physiological variation (^87^,^99^). Therefore, HRV is best regarded as complementary to CARTs, offering earlier and more prognostic information rather than a substitute tool in clinical practice (^87^).

Other methods for CAN assessment (^9^), include baroreflex sensitivity, muscle sympathetic nerve activity, plasma catecholamine measurement, and cardiac sympathetic imaging using nuclear medicine techniques. Their use is largely limited to research because of technical complexity, limited availability, and high cost (^9^,^87^).

The American Diabetes Association (ADA) recommends annual CAN evaluation in all individuals with T2D and in those with T1D of ≥ 5 years’ duration (^7^).

The Toronto Consensus (^9^), the ADA (^7^), the American Association of Clinical Endocrinologists (AACE)/American College of Endocrinology (ACE) (^100^) and the Italian Society of Diabetology (^43^) recommend screening for CAN, particularly in individuals with diabetes and long-standing disease, poor glycemic control, or other complications. All endorse symptom evaluation, but guidance on CARTs differs: they are considered the gold standard by the Toronto Consensus; recommended by the AACE/ACE and Italian Society; and regarded as optional by the ADA, mainly in symptomatic patients (*e.g.*, tachycardia, poor glycemic control or clinically suspected CAN), though potentially useful in asymptomatic individuals. The ADA favors a symptom-based approach for cost and feasibility reasons.

#### Management of CAN

At present, no disease-modifying therapy is available for CAN. Prevention strategies include lifestyle modifications, as demonstrated in the Diabetes Prevention Program (DPP) study (^101^), where participants engaging in ~150 min/week of exercise plus a low-fat diet had improved HRV and reduced incidence of T2D compared with metformin or placebo (^101^).

Several studies support the benefits of exercise on CAN, showing that regular physical activity (especially aerobic training) is associated with improved HRV (higher SDNN, RMSSD, pNN50, and HF; lower LF and LF/HF ratio), reflecting enhanced PNS activity and reduced SNS overactivity (^102^,^103^). However, data are limited and of low-quality in T2D without or with early CAN and scarce in established CAN or T1D (^8^).

Because individuals with diabetes and CAN are at increased risk of SMI, the ADA advises performing an exercise stress test before initiating an exercise program (^104^). Exercise prescriptions should also include safety precautions: avoid activities involving rapid postural changes in individuals with OH to reduce fainting risk; monitor intensity by HR reserve and perceived exertion in those with blunted HR response; and, in general, avoid hot environments and ensure adequate hydration (^104^).

Evidence suggests a stronger impact of glycemic control on CAN outcomes in T1D than in T2D. In T1D, the DCCT reported a 53% reduction in CAN incidence after 6.5 years of intensive insulin therapy, and the EDIC study confirmed a persistent protective effect for up to 14 years, despite subsequent convergence of A1c levels (^33^). In T2D, results are less consistent: the UKPDS demonstrated a 25% reduction in microvascular complications, but no clear reduction in CAN (^30^) and the VA Cooperative Study found no significant difference in CAN prevalence after two years of intensive therapy (^105^). By contrast, the Steno-2 trial (^106^) reported a 68% reduction in CAN incidence with a multifactorial strategy addressing glycemic, BP, lipid and albuminuria treatment, lifestyle modification, and smoking cessation, underscoring the importance of comprehensive risk factor management beyond glucose lowering. Accordingly, the ADA recommends early and intensive glycemic control to prevent CAN in T1D, a multifactorial approach targeting glycemia and other CV risk factors in T2D, and lifestyle interventions in individuals with prediabetes (^7^).

Currently, no antidiabetic drug has demonstrated consistent benefits for CAN. A small study demonstrated that metformin-related decrease in plasma free fatty acid and insulin resistance was associated with an improvement in sympathovagal balance (^107^). Although sodium-glucose cotransporter-2 inhibitors (SGLT2i) improve CV outcomes, their direct impact on autonomic dysfunction remains unclear. SGLT2i induces BP reductions without compensatory increase in HR in major trials, suggesting a SNS activity dampening (^108^). In the EMBODY trial (^109^), 96 patients with T2D after acute myocardial infarction treated with empagliflozin for 24 weeks demonstrated withingroup HRV improvements (higher SDNN and lower LF/HF ratio) and better HR turbulence. In contrast, the EMPA-HEART CardioLink-6 Holter analysis in 66 patients with T2D with stable coronary artery disease demonstrated no significant HRV changes after 6 months of empagliflozin versus placebo, suggesting no measurable impact on autonomic tone (^110^).

Studies with GLP1-RAs suggest a potentially unfavorable autonomic profile (^111^). Experimental data demonstrate increased HR and SNS activity with reduced HRV (^8^,^112^). This increase in HR may be explained by an action of GLP1 on receptors present on cardiomyocytes and an increase in SNS activity both directly and mediated by the GLP1-driven increase in endogenous insulin (^8^). However, clinical studies reported inconsistent effects of GLP1-RA on HRV, likely reflecting species-specific receptor patterns, agent differences, while not excluding direct effects on the sinus node (^8^). These findings should be weighed against the robust CV benefits of GLP1-RAs demonstrated in large clinical trials (^8^,^107^).

Pathogenesis-based therapies for CAN have yielded mixed results. Alpha-lipoic acid improved HRV in both T1D and T2D, via antioxidant and antiinflammatory effects (^113^,^114^). Aldose reductase inhibitors demonstrated inconsistent HRV benefits and potential adverse effects (^8^,^115^). Angiotensinconverting enzyme inhibitors and angiotensin receptor blockers produced similarly inconsistent findings (^8^,^9^). Cardioselective β-blockers may help restore sympathovagal balance and manage resting tachycardia (^116^). Overall, some agents appear promising, but require larger, long-term trials to establish efficacy.

Managing OH remains challenging. In individuals with OH, office BP assessment should emphasize postural changes from supine to standing rather than seated values, as these measurements guide therapeutic decisions and individualized target definition (^117^). First-line management is nonpharmacological and include: reducing or discontinuing aggravating medications, when possible (*e.g.*, tricyclic antidepressants, diuretics, vasodilators, α1-blockers) (^118^); dietary strategies such as increased fluid and salt intake to expand plasma volume (^9^,^118^) and rapid ingestion of 500 mL of water (^119^), if not contraindicate; small low-carbohydrate meals and alcohol restriction to minimize splanchnic postprandial blood pooling (^1^,^6^,^7^); lower limb strengthening exercises and counter-maneuvers (gradual positional changes, leg crossing and squatting.), while avoiding heat exposure and prolonged standing (^9^,^118^); elevation of head of the bed 10-30° at night to reduce nocturia and morning orthostatic intolerance (^9^,^118^); and compression garments to minimizing venous pooling in the legs and abdomen (^7^,^9^,^118^).

Pharmacological treatment for OH is reserved for refractory cases. Food and Drug Administration (FDA) approved options for symptomatic OH are midodrine, an α-1 adrenergic agonist that induces vasoconstriction and increased peripheral resistance; and droxidopa, a norepinephrine precursor that enhances SNS tone (^1^,^2^,^19^,^59^).

Fludrocortisone, a mineralocorticoid, expands plasma volume via sodium and water retention, but may induce supine hypertension, heart failure, edema and hypokalemia (^1^,^19^,^59^), requiring caution. Other less established agents include pyridostigmine, octreotide, desmopressin, and erythropoietin (^8^). All drugs should be initiated in the morning at low doses, titrated gradually, and avoided in the evening to minimize supine hypertension.

Treating supine hypertension is particularly difficult, as BP must be lowered without worsening OH. It is defined as SBP ≥ 140 mmHg and/or DBP ≥ 90 mmHg after 5 minutes in the supine position (^117^). Pharmacological therapy is usually reserved for more severe cases (SBP ≥ 180 mmHg or DBP ≥ 110 mmHg) (^118^). Short-acting antihypertensives given at bedtime, such as clonidine, captopril and losartan are recommended (^8^,^118^). The goal of therapy is symptom relief, prevention of syncope and falls, and preservation of functional independence, rather than strict BP normalization, and higher BP thresholds can be tolerated (^2^,^8^,^117^,^118^).

Detection of CAN in asymptomatic patients provides key information for risk stratification of diabetes complications, CV morbidity and mortality, and perioperative risk for major surgery (^8^,^9^). It also guides therapeutic strategies, helping to define individualized glycemic targets. In symptomatic patients, identification of clinical CAN enables tailored management of its consequences - such as tachycardia, non-dipping, nocturnal hypertension, and OH - and assists clinical decisions, including avoiding medications that prolong QT interval or impair sinoatrial node function, refraining from using seated BP as therapeutic target, incorporating 24hour BP monitoring, and developing a personalized, safe physical activity plan (^8^,^9^).

### Gastrointestinal autonomic neuropathy

The gastroenteropathy associated with diabetes may present with nonspecific despite burdensome symptoms (^17^). Autonomic neuropathy has an important role on its pathogenesis and may involve any part of the GI tract (^17^).

In a population study including 8,657 individuals, 453 (4.9%) with self-reported diabetes, symptoms of abdominal pain or discomfort (13.5% *vs.* 10.8%), early satiety (5.2% *vs.* 4.3%), postprandial fullness (8.6% *vs.* 5.2%), bloating (12.3% *vs.* 11.4%), heart burn (13.5% *vs.* 10.8%), nausea (5.2% *vs.* 3.5%), dysphagia (5.4% *vs.* 1.7%), vomiting (1.7% *vs.* 1.1%), diarrhea or constipation (15.6% *vs.* 10.0%), anal blockage (7.7% *vs.* 5.0%), fecal incontinence (2.6% *vs.* 0.8%), urgency (7.4% *vs.* 5.5%), esophageal symptoms (15.4% *vs.* 11.5%), upper dysmotility symptoms (18.2% *vs.* 15.3%) and any bowel symptom (26% *vs.* 18.9%) were significantly more frequent in individuals with diabetes than in controls, respectively (^120^). In diabetes, gallstones may be asymptomatic or cause discomfort or pain (^121^).

There is no clinically available method for screening GI neuropathy in diabetes. Therefore, active symptom inquiry is essential with exclusion of other GI disorders and drug-related side effects (^17^). Validated questionnaires are useful for deciding when to investigate GI autonomic neuropathy (^17^).

Esophageal symptoms, such as dysphagia and odynophagia, may occur in patients with diabetes, although dysmotility may be asymptomatic. Mechanical and infectious causes (*e.g.*, esophageal candidiasis) must be excluded (^17^) and treatment follows the same principle as for patients without diabetes. If dysmotility is suspected, patients should be instructed to drink water after taking oral medications to prevent pill esophagitis (^17^).

#### Gastroparesis

Gastroparesis is characterized by delayed gastric emptying in the absence of organic causes such as gastric or duodenal obstruction (^7^,^122^). It complicates glycemic management, contributes to glycemic variability or unexplained postprandial hypoglycemia due to a mismatch between nutrient absorption and insulin pharmacokinetics (^6^). It also leads to unpredictable drug absorption (^40^).

In 2020, a United European Gastroenterology (UEG) and European Society for Neurogastroenterology and Motility (ESNM) consensus on gastroparesis defined gastroparesis as a chronic condition, in which nausea and vomiting are cardinal symptoms, often accompanied by early satiety, epigastric pain, bloating, and postprandial fullness. Its symptoms may overlap with functional dyspepsia (^123^) and should be investigated (^122^,^123^).

In case of weight loss, eating disorders must be excluded (^123^). The impact of drugs, such as opioids, anticholinergics and incretins-based therapies on gastric emptying should also be considered, as they may precipitate or exacerbate symptoms (^7^,^124^).

Upper GI Endoscopy is mandatory to rule out mechanical obstruction (^122^,^123^) and mucosal disorders (^17^). Retained gastric food is not diagnostic of gastroparesis (^122^,^123^).

According to the American College of Gastroenterology (ACG) (^122^) and the ADA (^17^), gastric emptying scintigraph with a solid meal over ≥ 3 hours is the gold standard test for diagnosing gastroparesis. The stable isotope breath test is a reliable test (^17^,^122^). Similarly, the 2020 European Consensus (^123^) endorsed both methods as valid diagnostic tools. Wireless motility capsule testing may be considered (^122^), although not validated by the European consensus (overall agreement of 33%) (^123^). Radiopaque markers testing is not recommended by ACG (^122^).

Optimization of glycemic control before testing for gastroparesis (ideally between 70-180 mg/dL) is recommended. Testing should be avoided during hyperglycemia or hypoglycemia, since acute hyperglycemia delays gastric emptying, whereas hypoglycemia accelerates it (^125^). Medications that affect gastric emptying should be discontinued 48 hours before testing (^122^), and patients should abstain from smoking or alcohol (^17^,^18^,^121^).

Management of gastroparesis is challenging. Conservative and educational strategies should be implemented, including withdrawal of drugs that delay gastric emptying, dietary changes (small, frequent, low-fat, low-fiber meals, liquid or smallparticle diet) (^17^,^122^), avoidance of reflux-triggering foods and alcohol, and remaining upright for 1-2 hours after meals (^18^,^35^,^126^). Treatment may require antidiabetic therapy adjustment, such as reducing preprandial insulin dose or delaying its administration. Continuous subcutaneous insulin infusion therapy is an alternative in cases of marked glycemic variability (^18^,^35^,^126^).

Pharmacological therapy is reserved for refractory symptomatic cases (^122^). Metoclopramide is the only Food and Drug Administration (FDA) and European Medicines Agency (EMA) approved prokinetic, but its use should be limited to ≤ 3 months due to late-onset dyskinesia risk (^18^,^35^). Domperidone represents an alternative with fewer central nervous system (CNS) effects; however caution is required given its arrhythmogenic potential, also reported with metoclopramide (^17^,^35^,^122^). Erythromycin and other macrolides are limited to short-term use due to tachyphylaxis (typically after 2-4 weeks) (^17^,^35^). Serotonin 5-HT4 receptor agonists (*e.g.*, prucalopride) may also enhance gastric emptying (^122^,^123^).

For refractory nausea and vomiting, gastric electrical stimulation may be considered in non-opioid users (^122^), although not endorsed by all guidelines (^123^). Enteral feeding can be indicated in severe weight loss or malnutrition (^17^,^121^,^123^). Benefits of surgical interventions remain uncertain (^122^,^123^,^121^). Central modulators are not recommended for gastroparesis management (^122^,^123^).

#### Chronic constipation

The 2023 American Gastroenterological Association (AGA)/ACG guideline (^127^) made some useful recommendations on chronic idiopathic constipation (CIC) in adults, although not specifically associated with DAN. The guideline focused on otherwise healthy individuals and does not apply to children, pregnant women or individuals with opioid-induced constipation or malignancy (^127^).

Definition of functional constipation may vary in literature. According to the Rome IV criteria for adults (^128^), diagnosis requires symptoms for > 6 months, with at least two or more of the following manifestations occurring in at least 25% of defecations during the past three months: straining; hard or fragmented stools (Bristol 1-2); sensation of incomplete evacuation; sensation of anorectal obstruction; manual maneuvers to facilitate defecation; less than three spontaneous bowel movements per week; loose stools should rarely occur without the use of laxative and criteria for irritable bowel syndrome are not met (^128^).

A detailed clinical history, including dietary habits, and complementary tests are required to exclude other causes of constipation, such as medications, mechanical obstruction, metabolic disorders, myopathies and neurologic diseases (^17^,^129^).

The panel suggested the use of fiber supplementation with low certainty of evidence (^127^). Adjusting a low fiber intake through diet or supplements can be used as first-line therapy.

Only psyllium appears effective, while data on bran and inulin supplementation are limited. Adequate hydration should accompany fiber intake (^127^).

Polyethylene glycol (PEG) is strongly recommended as an osmotic laxative after or combined with fiber supplementation, with moderate certainty of evidence. Evidence supports PEG efficacy in CIC for up to six months, while long-term efficacy and safety data remain unavailable (^127^).

Magnesium oxide was suggested with very low certainty of evidence (^127^), however, should be avoided in patients with renal dysfunction due to the risk of hypermagnesemia. Lactulose may be considered in adults unresponsive to fiber and overthe-counter therapies, although the certainty of evidence was also very low (^127^).

Short term use (≤4 weeks) of stimulant laxatives such as bisacodyl or sodium picosulfate as rescue therapy had a strong recommendation with moderate certainty of evidence. Long-term use is probably appropriate, but lacks supporting data (^127^). Senna was suggested with low certainty of evidence, and long-term safety data are also missing (^127^).

Patients unresponsive to over-the-counter agents, other agents such as lubiprostone, plecanatide, linaclotide and prucalopride had specific recommendations by the panel (^127^). For refractory cases, management should follow specific guidelines (^129^).

#### Chronic diarrhea

Diabetic diarrhea is typically chronic, painless, and watery, often occurring at night (^121^). A detailed clinical history is essential, with careful evaluation of medications use such as metformin, GLP1- RAs, lipase inhibitors, sugar alcohols (*e.g.*, sorbitol) (^17^). The temporal relationship between drug initiation and the symptom onset must be well investigated.

As diabetic diarrhea is an exclusion diagnosis, the 2019 AGA clinical practice guideline’s recommendations for functional diarrhea (^130^), are useful to exclude other causes of chronic diarrhea in immunocompetent patients (^130^). They include fecal calprotectin or lactoferrin as screening tools for inflammatory bowel disease, testing for *Giardia* and screening for celiac disease (^130^).

Delayed small intestine transit may lead to small intestinal bacterial overgrowth (SIBO), a cause of diarrhea. Colonic involvement is also frequent (^131^).

Management of lower GI symptoms also includes glycemic control and dietary adjustments. Antidiarrheal agents (*e.g.*, loperamide) may be effective but may worsen constipation and upper GI symptoms. SIBO is commonly treated with antibiotics (^18^,^35^,^121^).

### Genitourinary autonomic neuropathy

Voiding is a coordinated process requiring sustained detrusor contraction, relaxation of the bladder neck, sphincter and urethra, and absence of outlet obstruction (^132^). These functions are regulated by the ANS through a neural network involving peripheral nerves, spinal cord, and CNS (^133^,^134^). The SNS facilitates bladder storage by inhibiting the detrusor activity and stimulating the trigone and proximal urethra contraction (^134^), while the PNS mediates voiding through detrusor contraction and sphincter relaxation (^4^).

In diabetes, GU autonomic neuropathy manifests mainly as sexual dysfunction and LUTS (^7^), after exclusion of organic causes such as hypogonadism, medication effects, prostatic disease, and pelvic disorders (^7^,^126^,^135^-^137^).

#### Sexual dysfunction

In men with diabetes, ED occurs earlier, is more severe, and often less responsive to treatment than in the general population (^138^). Male dysfunction may also involve orgasmic, ejaculatory, and libido abnormalities (^21^).

In T1D, good glycemic control reduces ED incidence, while evidence in T2D is less consistent (^7^). Lifestyle measures such as weight optimization, BP and lipid control, and smoking cessation can improve ED (^7^). First-line therapy includes phosphodiesterase type 5 inhibitors (tadalafil, sildenafil, vardenafil). Intracavernosal or intraurethral prostaglandins, vacuum devices, and penile prostheses are reserved for advanced cases (^7^,^138^,^139^).

In women, sexual dysfunction manifests as reduced libido, arousal and lubrication, as well as dyspareunia (^7^,^138^). Management includes glycemic control, treatment of urinary/genital infections, lubricants or moisturizers, pelvic floor physiotherapy, and psychological support (^19^,^140^).

#### LUTS

Diabetic cystopathy is initially characterized by reduced bladder sensation and increased capacity, often accompanied by overactive bladder (OAB) symptoms including urinary frequency, urgency and UI (^140^).

With progression, detrusor impairment leads to incomplete emptying and underactive bladder, culminating in combined storage and voiding dysfunction (^140^,^141^). This manifests as delayed initiation of micturition, reduced bladder sensation, increased post-void residual (PVR), urinary retention, overflow incontinence, and recurrent UTI (^140^).

Additional LUTS include nocturia, weak urinary stream and hesitancy (^7^,^21^). In men, concomitant benign prostatic hyperplasia (BPH) is common and may overlap symptoms with LUTS (^140^,^142^).

Diagnosis of LUTS is predominantly clinical (^1^,^7^). The European Association of Urology (EAU) (^136^,^143^) and the National Institute for Health and Care Excellence (NICE) (^137^,^144^) guidelines recommend initial evaluation with a comprehensive medical history, validated symptom questionnaires, and a ≥ 3 days bladder diary documenting volume and timing of voids. These recommendations apply to the general population, not specifically to individuals with diabetes.

Individuals with diabetes with recurrent UTI, pyelonephritis, UI or a palpable bladder should undergo evaluation of urinary dysfunction, including urinalysis and assessment of renal function (^4^).

When LUTS are associated with UTI, reassessment is required after antibiotic treatment (^143^). Urinary tract or prostate ultrasound with PVR measurement may be indicated (^136^,^137^,^143^,^144^). A complete urogynecological examination is advised to rule out structural abnormalities, along with a pelvic floor assessment to evaluate muscle contraction (^135^,^138^,^144^). Urodynamic testing and cystoscopy are reserved for selected cases as indicated by specialist (^4^,^136^,^143^,^144^,^144^).

Treatment of LUTS includes behavioral, pharmacological, and, in selected cases, surgical approaches (^140^).

Behavioral strategies involve fluid restriction at night to minimize nocturnal polyuria, moderation of caffeine and alcohol intake, voiding techniques (relaxed or double voiding, urethral milking), bladder training, distraction techniques for urgency control, constipation management (^136^,^137^), and, in women, weight loss and pelvic floor muscle training (^143^,^144^). Intermittent catheterization may be required in significant voiding dysfunction with severe upper urinary tract involvement to preserve renal function, and medication review is advised to discontinue drugs that worsen LUTS, particularly diuretics and drugs such as amitriptyline (^136^,^140^,^145^,^146^).

Pharmacological treatments are recommended for bothersome moderate-to-severe LUTS, with comorbidities, concomitant treatments and sex-specific factors guiding treatment choice (^136^,^137^,^143^,^144^).

In men, α1-adrenergic antagonists (doxazosin, tamsulosin, alfuzocin, terazocin, silodosin and naftopidil) provide rapid relief of storage and voiding symptoms (^136^,^137^), but may cause asthenia, dizziness, and OH, requiring caution in patients with CAN, CVD or concomitant vasoactive drugs (^137^,^147^,^148^).

5α-reductase inhibitors (dutasteride and finasteride) may be considered in prostatic enlargement, elevated PSA (Prostate-Specific Antigen), or other progression risk factors (*e.g.*, older age), as they reduce prostate volume (^137^,^148^) and the risk of acute urinary retention (^148^).

Combination therapy offers superior efficacy but higher adverse events, and should be reserved for moderate-to-severe LUTS with progression risk (^137^,^148^). For patients with refractory symptoms, the EAU and NICE guidelines provide specific recommendations (^137^,^143^,^144^,^148^).

According to the EAU guidelines, direct (bethanechol, carbachol) and indirect (neostigmine, distigmine) parasympathomimetics are not recommended for underactive bladder (^148^).

In cases of OAB and urgency UI/storage symptoms, antimuscarinic (*e.g.*, oxybutynin, tolterodine and solifenacin) or β3-adrenergic agonists (vibegron or mirabegron) are the main options when conservative measures fail (^137^,^143^,^144^,^148^). Antimuscarinics may cause dry mouth, constipation and cognitive effects, requiring caution in older or cognitively impaired patients (^144^,^148^).

Individuals with diabetes also have a higher risk of asymptomatic bacteriuria (^149^), but screening or treatment is not recommended by the Infectious Diseases Society of America guideline (^150^), given the absence of benefit and risk of antimicrobial resistance and *Clostridioides difficile* infection (^151^).

SGLT2i may increase the risk of urogenital infections (particularly fungal) (^152^-^154^), and enhance urine output, potentially worsening LUTS such as frequency, urgency and nocturia (^155^,^156^). Evidence on the safety and efficacy of SGLT2i in individuals with structural or functional urinary tract abnormalities remain limited (^157^). Thus, LUTS severity should be assessed before SGLT2i initiation, and treatment individualized, particularly in severe cases with indwelling catheters, ileal conduits, recurrent UTI, or high PVR, with close follow-up to minimize complications (^157^,^158^).

## CONCLUSION

In conclusion, the ANS regulates multiple organs and systems through an extensive network of afferent and efferent nerve fibers. DAN is a heterogeneous condition with a broad spectrum of manifestations - including CV, GI, and GU involvement - progressing from early, asymptomatic small-fiber involvement to severe, advanced manifestations. DAN is a frequent but underrecognized complication, with substantial impact on morbidity and mortality. Early diagnosis is essential for CV risk stratification, optimization of therapeutic targets, and timely symptom management. Although no disease-modifying therapies are currently available, management strategies are primarily symptomatic, aiming to alleviate clinical manifestations. Current recommendations emphasize glycemic control, comprehensive CV risk management, and individualized treatment of clinical manifestations to improve quality of life and potentially influence long-term outcomes.

## Data Availability

datasets related to this article will be available upon request to the corresponding author.
